# Specific but not general declines in attention and executive function with aging: Converging cross-sectional and longitudinal evidence across the adult lifespan

**DOI:** 10.3389/fpsyg.2023.1108725

**Published:** 2023-03-15

**Authors:** Shulan Hsieh, En-Ho Chen

**Affiliations:** ^1^Cognitive Electrophysiology Laboratory: Control, Aging, Sleep, and Emotion (CASE), Department of Psychology, National Cheng Kung University, Tainan, Taiwan; ^2^Institute of Allied Health Sciences, National Cheng Kung University, Tainan, Taiwan; ^3^Department of Public Health, National Cheng Kung University, Tainan, Taiwan

**Keywords:** alerting, orienting, conflict, stopping, memory updating and switching

## Abstract

**Objective:**

Attention and executive function (EF) are vulnerable to aging. However, whether all these functions generally decline with aging is not known. Furthermore, most evidence is based on cross-sectional data and fewer follow-up data are available in the literature. Longitudinal follow-up studies are necessary to characterize individualized and precise changes in cognitive function. Additionally, relatively few aging studies have included middle-aged adults to examine age-related differences in attention and EF. Therefore, this study aims to examine whether general or specific attention and EF decline with aging from adulthood to old age by combining cross-sectional and longitudinal follow-up approaches.

**Methods:**

This study recruited 253 participants aged 20 to 78  years. passing a prescreening procedure (see main text for detail) for the baseline session, and 123 of them were invited to return 1 ~ 2  years after their first visit to participate in the follow-up session. The participants completed a series of attention and EF tasks at both the baseline and follow-up sessions, which measured alerting, orienting, conflict control, stopping, memory updating, and switching abilities. We applied linear and nonlinear regression models to evaluate the cross-sectional age effect on attention and EF and employed a modified Brinley plot to inspect follow-up performance against baseline in attention and EF.

**Results:**

The results of cross-sectional data showed that older adults exhibited decreased efficiency in alerting, stopping, and memory updating but paradoxically increased efficiency in conflict control and switching abilities and no changes in orienting efficiency with age. However, the results of longitudinal data showed that only alerting and memory updating continued to show decreased efficiency. Furthermore, conflict control and switching showed increased efficiency with aging, whereas the orienting network, and stopping no longer showed decreased efficiency.

**Conclusion:**

Thus, converging the cross-sectional and longitudinal data showed that the alerting and memory updating function exhibited the most robust deficit with age (cross-sectional) and aging (longitudinal). Alerting and memory updating abilities are crucial survival skills for human beings. Therefore, developing methods to prevent and improve an individual’s alertness and working memory ability is an important practical issue in aging research.

## Introduction

When an individual ages, the stereotypical behavioral change is that the individual becomes slower, less dexterous, and less ingenious. However, the literature has shown that not all cognitive functions decline with age. Some studies based on cross-sectional databases reported that crystallized intelligence, such as world knowledge, simple arithmetic calculation ability, vocabulary, and verbal ability, did not decline with age, whereas fluid intelligence, such as processing speed, working memory, and the ability to reason and think flexibly, declined with age (e.g., Park et al., 2002; Schaie, 2013). However, even fluid intelligence has not been unequivocally shown to have general age-related declines. For example, although most studies have reported a general deficit in executive function (EF, known to be highly related to fluid intelligence; van Aken et al., 2016) with age (West, 1996; Raz, 2000; Braver and West, 2008), other researchers have discovered that only some aspects of EF decline with age. For example, one earlier representative review study by Verhaeghen (2011, 2014), who conducted meta-analyses on aging and executive control, showed that EF is not a unitary construct that is generally affected by age but can be separated into different component processes, such as resistance to interference, coordinative ability, task shifting, and memory updating, which are differentially affected by age. Rey-Mermet and Gade (2018) further classified various forms of inhibition (one form of EF) that were measured by multiple tasks, such as the color Stroop, flanker, Simon, stop-signal, go/no-go, global–local, and n-2 repetition effect costs in task switching. They also found that not all forms of inhibition, only those measured by go/no-go and stop-signal tasks, declined with age. Therefore, based on these prior studies, research addressing age-related effects on EF should consider its multicomponent characteristics.

In addition to EF, attention has also been considered age-sensitive and plays a critical role in daily activities for human beings, such as driving, sports, attending a lecture, or reading a book without repeatedly being distracted by the sights, sounds, and smells of the environment. However, attention likewise is by no means a unitary concept but can be manifested in various aspects (James et al., 1890; Parasuraman and Davies, 1984), such as alerting, orienting, selective, sustained, divided, shifting, attentional control, etc. Over the years, the attention network test (ANT), developed by Posner and Petersen (1990) and Fan et al. (2002), has been widely used to capture group and individual differences in attention and EF, which contains three networks, i.e., alerting, orienting, and executive control.

By means of the ANT, Veríssimo et al. (2022) recently examined age effects on alerting, orienting, and executive control abilities. Their results showed that while the efficiency of the alerting network decreased with age, the efficiency of orienting and executive control networks paradoxically increased. Although, Veríssimo et al. (2022) examined a large sample to show age-related differences in attention and EF, the EF captured by ANT is just one example. The EF network in the ANT focused only on one form of inhibitory function, i.e., the resistance to a distractor interference as indexed by the flanker effect. However, as mentioned above, inhibitory function can also refer to the inhibition of a prepotent response and the inhibition of an ongoing response (See Barkley, 1997; Rey-Mermet and Gade, 2018). In addition, EF likewise refers not only to an inhibitory function but also to memory updating and shifting ability, as mentioned above (Verhaeghen, 2011, 2014; see also Miyake et al., 2000). Therefore, this study incorporated various EF tasks to re-examine age-related effects on attention and EF. These additional functions included memory updating measured by a 2-back task (Gazzaniga et al., 2009) and shifting ability measured by a task-switching paradigm (see reviews by Kiesel et al., 2010; Vandierendonck et al., 2010). In addition, we added a stop-signal task (Band et al., 2003) to tap into another form of inhibitory function, i.e., the inhibition of an ongoing response.

Furthermore, the aging literature has emphasized that the results from a cross-sectional approach may differ from those of a longitudinal approach, where the former confounds the cohort effect (Hedden and Gabrieli, 2004; Rugg, 2016; Hsieh and Yang, 2021). In addition, the phenomenon of specific but not general deficits in EF can also be linked to the concept of “cognitive reserve” (Stern, 2002, 2009). Individuals’ life experience, such as educational or occupational attainment, can impart reserve against brain atrophy, allowing some people to maintain function longer than others (Steffener et al., 2014). Thus, individual differences in aging trajectories should not be overlooked, which requires a longitudinal approach to delineate the patterns. Therefore, this study used both cross-sectional and longitudinal approaches. Additionally, as indicated by Ferguson et al. (2021), relatively few aging studies have included middle-aged adults in investigations of age-related differences in EF, so this study recruited young adults (age > = 20 years) through old age to obtain a more informed understanding of attention and EF across the adult lifespan. We hypothesized that not all forms of attention and EF would be uniformly but selectively affected by age (cross-sectional) or aging (longitudinal). Furthermore, cohort effects and individual differences may exist across different tasks, resulting in different patterns between cross-sectional and longitudinal data.

## Method

### Participants

Participants were recruited from the community *via* leaflets, social networks, bulletin board system (BBS), and bulletin boards at the National Cheng Kung University (NCKU), Tainan, Taiwan, as part of the Cognitive Aging project. The project’s research assistants (RAs) contacted the potential participants who were registered online *via* a Google form provided by the abovementioned advertisements to participate in the study. During the telephone contact, RAs prescreened the registered individuals based on the criteria of being right-handed, native speakers of Chinese, self-report of normal or corrected-to-normal vision, having no color blindness, no history of neurological disorders (e.g., brain tumor, stroke, concussion, etc.), psychiatric disorders (e.g., depression, anxiety, schizophrenia, bipolar disorder, obsessive–compulsive disorder, etc.), cancer or metastatic cancer, or severe cardiovascular disease (note, treated hypertension was not excluded) and then invited those potentially qualified participants to the lab located at the NCKU, Tainan, Taiwan, to complete the questionnaires and computerized cognitive tasks (described in the procedure section). The total invited sample included 253 participants (mean age = 46.15 ± 16.52 years; age range = 20–78, female/male = 129/124) for the first visit (as baseline session). One hundred and twenty-three participants were invited to return 1 ~ 2 years after their first visit to participate in the follow-up session (mean age = 50.65 ± 16.73; age range = 22–80, female/male = 56/67). However, not all these participants’ data were subsequently analyzed, and analysis depended on how well the participants performed each of the computerized cognitive tasks (i.e., performance accuracy <75%). Additionally, some of the participants could be further excluded because they were found to have health problems when they visited the lab based on the responses in the Demographics and Health Status Questionnaire (modified and translated from the questionnaire used in Karayanidis’s Agility project) and two other screening questionnaires, i.e., the Beck Depression Inventory (BDI)-II (Beck et al., 1996; Chinese version by Lu et al., 2002) and Montreal Cognitive Assessment (MoCA; Nasreddine et al., 2005; Chinese version by Tsai et al., 2012). That is, if the participants reported health problems on the Demographics and Health Status Questionnaire that they did not self-disclose during the initial phone contact or if their MoCA or BDI scores did not pass the screening criteria (i.e., MoCA <26; BDI-II > 13), they were further excluded from subsequent data analyses. The remaining total number of participants for each of the cognitive tasks will be reported in the ‘Results’ section. We also used the Grooved Pegboard Test (GPT; Merker and Podell, 2011) to evaluate participants’ fine motor-control function, such as the dexterity of both the dominant and non-dominant hands.

### Ethics statement

The study protocol was approved by the human research ethics committee (NO# 104–004) at the National Cheng Kung University, Tainan, Taiwan. Participants received detailed information about the experimental procedure, risks, and benefits. They were asked to sign the informed consent form before any data acquisition. Participants received monetary compensation after completing all sessions. If they did not complete the assessment, partial monetary compensation was given based on the participation hours.

### Procedure

Participants completed a session of approximately 3 h. Each session included the assessments of the Demographics and Health Status Questionnaire, the Beck Depression Inventory (BDI)-II (Beck et al., 1996; Chinese version by Lu et al., 2002), Montreal Cognitive Assessment (MoCA; Nasreddine et al., 2005; Chinese version by Tsai et al., 2012), Grooved Pegboard Test (GPT; Merker and Podell, 2011), Attention Network Test (ANT; Fan et al., 2002, 2005), stop-signal task (SST; Logan and Cowan, 1984), 2-back task (Jaeggi et al., 2008), and task-switching task (TSWT; Karayanidis et al., 2011). Note, participants were able to rest between each cognitive task to avoid tiredness and transfer effects. Practically, if they could not complete all the assessments including the three cognitive tasks in a day, they would be allowed to make an appointment for another day (no more than 1 week apart) to complete the rest of the assessments. None of the participants reported here required an additional day to complete the assessments.

### Computerized cognitive tasks for measuring attention and EF

#### General instruments for visual presentation and laboratory settings

The visual stimuli used in the following computerized cognitive tasks were programmed using Presentation or E-Prime software and were displayed on a 17-inch monitor with 1,024 × 768 resolution. Participants were seated in a comfortable chair in a sound-attenuated room with dim light and a temperature of 25 degree Celsius, facing the computer screen at approximately 70 cm to perform each of the four computerized cognitive tasks (ANT, SST, 2-back task, and TSWT).

##### Attention network test

In this ANT (adapted from Fan et al., 2002, 2005), participants were instructed to fixate on a central cross for the entire experiment. The stimuli consisted of a row of five horizontal black arrows that appeared in either the upper or lower part of the screen and pointed either to the left or right. The target central arrow was pointed either in the same direction as the other four flanking arrows (congruent condition) or in the opposite direction (incongruent condition). Each trial started from a central fixation cross presented for a random duration of 400 ~ 1,600 ms (fixation duration, FD). In 1/4 of the trials, the stimulus (response waiting time less than 1,700 ms) was preceded by a visual cue (an asterisk; presentation duration of 100 ms) that appeared at the central fixation cross ‘+’ (central-cue condition; presentation duration of 400 ms). In another 1/4 of the trials, the stimulus was preceded by a visual cue that was presented above or below the central fixation cross (at the same location as the stimulus would appear) (spatial cue condition) and indicated that the stimulus would appear in the upper or lower part of the screen, respectively. In another 1/4 of the trials, the stimulus was preceded by two visual cues that were simultaneously presented above and below the central fixation cross (double-cue condition). In the remaining 1/4 of the trials, the stimulus was not preceded by any visual cue (no-cue condition). Participants were asked to determine the direction of the central arrow of the stimulus by pressing one of the two buttons on a computer mouse as quickly and accurately as possible. The waiting deadline for the button response was 1,700 ms. The next trial started after 3,500 ms (response time to target – FD). Performance scores for each of the three attentional networks were measured separately as follows.

The alerting score was calculated by subtracting the mean reaction time (RT) of the double-cue condition from the mean RT of the no-cue condition. The orienting score was calculated by subtracting the mean RT of the spatial cue condition from the mean RT of the center cue condition. The conflict (executive control) score was calculated by subtracting the mean RT of all congruent conditions across all cue types from the mean RT of the incongruent conditions. The entire experiment lasted for approximately 30–40 min.

##### Stop-signal task

This SST was modified from the paradigm of Logan and Cowan (1984), and the program was provided by Frini Karayanidis’s laboratory. The computer screen’s background was white, and the target stimulus was black. The target stimulus “O” or “X” was presented in the center of the screen for 100 ms (2 cm in size with a visual angle of 0.64°). Participants were instructed to look at the stimulus shown on the computer screen and press the “z” or “/” button corresponding to the target “O” or “X” with their left and right index fingers, respectively. An auditory stop signal was presented for 100 ms at a frequency of 1,000 Hz. The stop-signal delay (SSD) varied depending on the participant’s response to the stop trials, and the SSD for each stop trial was selected from one of two interleaved staircases, each starting with SSD values of 150 and 350 ms. If participants stopped successfully, the SSD would increase by 50 ms in the next stop trial; otherwise, if they failed to stop, a decrease of 50 ms was included in the next stop trial (SSD range, 0–800 ms). This staircase procedure ensured that the participant’s likelihood of stopping occurred approximately 50% of the time. The interstimulus interval (ISI) varied from 1,300 to 4,800 ms, and the stop-signal RT (SSRT) was calculated by subtracting the median SSD from the median RT of the go trials. A complementary calculation method of SSRT was also used, that is, the integration method (Logan and Cowan, 1984; Band et al., 2003; Verbruggen and Logan, 2008, 2009). For each SSD, the probability of responding to stop signals was determined. If an SSD of 50 ms resulted in a 25% error rate (stop-signal response [failed to inhibit] trials), the end of the stop process should be at a point equal to 25% of the GO RT distribution. If this point was 300 ms, the observed SSRT would be (300–50) = 250 ms. This procedure was repeated for each SSD (condition-based) for each participant. The mean SSRT was the average of these SSRTs estimated at various points.

The experiment included two blocks of practice. In the first practice block, a “beep” sound (1,000 Hz for 100 ms) occasionally occurred in the background, and participants were instructed to respond to the stimulus as soon and as accurately as possible and ignore this background sound. In the second practice block, participants were instructed to stop their reaction immediately whenever they heard the stop signal of a “beep” sound following stimulus onset. They were told not to slow their reaction to waiting for the stop signal to occur. After the practice, the formal experiment commenced, and all settings and rules were the same as those for the second practice block. The formal experiment included five blocks (140 trials per block, 40 of them stop trials). The completion time was approximately 30 min, including instruction and practice time.

##### 2-back task

The 2-back task is a continuous performance task that is commonly used as an assessment to measure a part of working memory (originally developed by Jaeggi et al., 2008). Participants would see a 3-by-3 grid in every trial, and one of the grids would be filled with a blue color. The blue grid would appear randomly in any of the 3-by-3 grids. Participants in a 2-back working memory test were instructed to compare whether every current blue grid’s location was the same as or different from the location of the blue grid 2 turns back. If they were at the same location, participants should press the “F” button with their left index finger, and if they were at different locations, participants should press the “J” button with their right index finger (counterbalanced across participants). The stimulus was presented for 500 ms, and participants had a 2,000-ms interstimulus interval (ISI) to respond. Participants went through one block of practice with feedback and three blocks of the formal experiment (21 trials per block). We calculated the performance sensitivity (d’) as a working memory updating index, which was based on the hit rate (H) and false alarm (F) rate. We first transformed the raw data into z scores. The formula is as follows: d’ = Z(Hit) – Z(False alarm). Larger values of d’ indicate a higher working memory updating ability.

##### Task-switching task

A cued-target TSWT modified by Karayanidis et al. (2011) was used (the original program was provided by Frini Karayanidis’s laboratory). Two types of color cues informed the forthcoming task type (informed task conditions), a hot color (e.g., orange, red) and a cold color (e.g., green, blue), associated with an Arabic number or a Chinese letter classification task, respectively. The cue color was never repeated in successive trials to reduce the effects of repeating a physically identical cue. Stimuli consisted of an incongruently mapped bivalent Chinese letter–Arabic number pair (e.g., 甲4 or 4甲) or a neutral pair (e.g., 丙% or #丙). Chinese letters refer to the eight Chinese letters (1^st^ half,「甲」, 「乙」, 「丙」, and「丁」, 2^nd^ half,「戊」, 「己」, 「庚」, and「辛」) derived from the system of the Ten Celestial Stems (i.e., Tiangan). Participants were required to respond using the left and right index fingers mapped to the first-half/s-half for the Chinese-letter tasks and odd/even for the Arabic-number tasks. Cue-task mapping and hand-task mapping were counterbalanced across the participants. On mixed-task blocks, the switching probability was 50% with no more than four mixed-repeat or switch trials in succession. In addition to the color cue conditions, in this study, we added neutral cue conditions (i.e., noninformed task conditions) in which the cues were drawn in black to not indicate the forthcoming task type, whereas instead, the stimuli were drawn in colors, either hot (red/orange) or cold (blue/green), thereby indicating the task to act upon.

Each trial consisted of a cue (either in color or black) and a target. A cue (‘+’) was presented for 600 ms followed by a cue-target interval of 1,000 ms and a target, which was presented for 5,000 ms or until a response was made. The interval between a response and the next target (response-target interval) was 1,600 ms. Participants were instructed to respond as quickly and as accurately as possible. Each error was followed by immediate auditory feedback, and the next trial was delayed by 1,000 ms. The mean RT and error rate feedback were provided after each block of trials.

The TSWT included task practice and formal experiment sessions. A practice session contained the following types of blocks: (1) one single-task block of numbers for 16 trials; (2) one single-task block of Chinese letters for 16 trials; (3) two mixed-informed task blocks (e.g., each trial was preceded with a color cue) for 32 trials per block; and (4) two mixed noninformed task blocks (e.g., each trial was preceded with a neutral color [black] cue) for 32 trials per block. The formal experiment consisted of 12 blocks: (1) two single-task blocks of numbers for 70 trials per block; (2) two single-task blocks of Chinese letters for 70 trials per block; (3) four mixed-informed task blocks for 70 trials per block; and (4) four mixed noninformed task blocks for 70 trials per block. The entire experiment lasted for approximately 30–40 min.

Switch cost (i.e., local switch cost) was calculated by subtracting the mean RT of the repeat trials in the mixed-task blocks from the mean RT of the switch trials in the mixed-task blocks. Please note that any trial immediately following an error was excluded from the analyses since it could not be classified as a switch or repeat trial condition.

## Statistical analysis

### Demographic information’s statistical analyses: Comparison between baseline and follow-up

We performed paired-*t* tests with a significant criterion of *p* < 0.05 using Statistical Program for Social Sciences, version 22.0 (SPSS) on each of the participant’s age (years), education (years), physical activity (level^1^), GPT performance (the averaged time spend by both hands^2^), MoCA (Likert-scale points), and BDI-II (Likert-scale points) between the baseline and follow-up sessions. We also performed the Wilcoxon signed rank tests between the two sessions for the categorical variables, such as annual income^3^ and occupational class^4^ (see Table 1^5^).

**Table 1:** Demographic information for all participants recruited in the baseline and follow-up sessions, and the statistical tests between the two sessions, if applicable.

	All (*n* = 253)	Subsample (*n* = 123)		
	Baseline (*n* = 253)	Baseline (*n* = 123)	Follow-up (*n* = 123)	Paired-*t*, *p*
*Age (year)*	46.15 (16.52)	48.87 (16.69)	50.65 (16.73)	−46.24, *p* < 0.00
Range (year)	20–78	20–78	22–80	–
*Education (year)*	14.88 (2.53)	14.85 (2.52)	14.84 (2.52)	0.00, *p* = 1
Range (year)	6–23	6–21	6–21	–
*Gender (F/M)*	129/124	56/67	56/67	–
*Physical activity (level)*	3.12 (1.47)	3.16 (1.51)	3.57 (1.47)	−2.81, *p* < 0.01
*GPT (seconds)*	78.68 (20.71)	76.81 (14.90)	77.81 (18.62)	−1.10, *p* = 0.27
*MoCA*	27.78 (1.90)	27.69 (1.85)	28.93 (1.26)	−7.44, *p* < 00
*BDI-II*	5.64 (4.72)	4.92 (4.12)	5.36 (5.91)	−0.87, *p* = 0.39
*Annual Income (NTD)*	*n = 253*	*n = 123*	*n = 123*	*Signed-rank test*
Not available	68	36	43	p = 0.89
Low	83	37	25	
Middle	62	26	33	
High	40	24	22	
*Occupational class*	*n* = 253	*n* = 123	*n* = 123	Signed-rank test
No job	128	63	69	*p* = 0.12
Light workload	58	29	23	
Moderate workload	59	28	30	
Heavy workload	8	3	1	

GPT, Grooved Pegboard Test; MoCA, Montreal Cognitive Assessment; BDI-II, Beck Depression Inventory-II; F, Female; M, Male; the values in brackets indicate standard deviations; Note: Physical activity was evaluated using a 5-point scale from 1 (rarely engage in physical activity) to 5 (participate in aerobic exercise, more than 3 h per week). Low income: under 120,000 NTD per year; Middle income: 120,000 ~ 480,000 NTD per year; High income: above 480,000 NTD per year; No job: student, retired, housekeeping, etc.; Light workload: Office worker such as manager, designer, computer engineer, clerical work; Moderate workload: Sales staff, long-term standing, and walking needs such as business, teachers, doctors, nurses; Heavy workload: Manual labor work such as laborer.

### Two sets of cross-sectional data analyses: One baseline and one follow-up test session

We plotted each participant’s performance score data point on each of the cognitive tasks as a function of age collected at baseline and follow-up test sessions separately.

For the ANT analyses, we first log-transformed all the RT data before statistical analyses. Both linear and nonlinear mixed-effects regression models were applied by the lme4 package (version 1.1–30) in R (version 4.2.1) (Bates et al., 2014, 2015). The fixed effects were age (in years; continuous), cue type (center, double, no cue, and spatial), and flanker type (congruent and incongruent). The interactions among these predictors were also included as predictors, i.e., age x cue type, cue x flanker type, flanker type x age, and the interactions of interest that allow estimating the effect of age on the three attentional networks. In addition, education years, gender, and trials in the experimental order were included as covariates to control for correlations between age and these variables. The contrasts for cue type in the three ANT network models were as follows: alerting effect: center = 0, double = −0.5, no cue = 0.5, and spatial = 0; orienting effect: center = 0.5, double = 0, no cue = 0, and spatial = −0.5; and conflict effect: congruent = −0.5 and incongruent = 0.5. All control variables were set as gender: female = −0.5 and male = 0.5, and the continuous variables, such as age, education years, and trials in the experimental order, were subtracted from their mean values to be in the center. A random effect on the participant’s intercept and flanker type random slope by the participant was included to allow the flanker effect to be randomized in the model.

For the SSRT and 2-back d’ analyses, we employed both linear and nonlinear regression models and added age as a predictor, with education years and gender as covariates.

For the switch cost analyses, we first log-transformed all the RT data before statistical analyses. Both linear and nonlinear mixed-effects regression models were applied. The fixed effects were age (in years; continuous) and trial type (repeat, switch). The two-way interaction among these predictors was also included as predictors, i.e., age x trial type. In addition, education years, gender, and trials in the experimental order were included as covariates to control for correlations between age and these variables. The contrasts for the models were as follows: switch cost: repeat = −0.5 and switch = 0.5. All control variables were defined as gender: female = −0.5 and male = 0.5, and the continuous variables, such as age, education years, and trials in the experimental order, were subtracted from their mean values to be in the center. A random effect on the participant’s intercept was included to allow the switch effect to be randomized in the model.

### Longitudinal analysis: Modified Brinley plot analysis by plotting the baseline session performance against the follow-up session performance

To identify possible significant changes in performance from the baseline to follow-up session, we adopted a modified Brinley plot analysis (Jacobson and Truax, 1991; Rucklidge and Blampied, 2011). When we applied the modified Brinley plot to interpret the follow-up performance, we assigned the X-axis to be an individual’s performance in the baseline session, and the Y-axis was the performance in the follow-up session; we then plotted each individual’s data point in the X-Y plane. The rationale is that the data points plotted above the diagonal line represent individuals whose follow-up performance was larger than that in the baseline session. However, a larger follow-up performance than baseline performance (i.e., data points above the diagonal line) reflected an increase or decrease in performance efficiency depending on each measure’s characteristics (see the results section for details). For example, for an alerting score, an increase in scores indicates better performance, whereas for a conflict score, an increase in scores indicates worse performance.

## Results

### Participants

**Figure 1:**
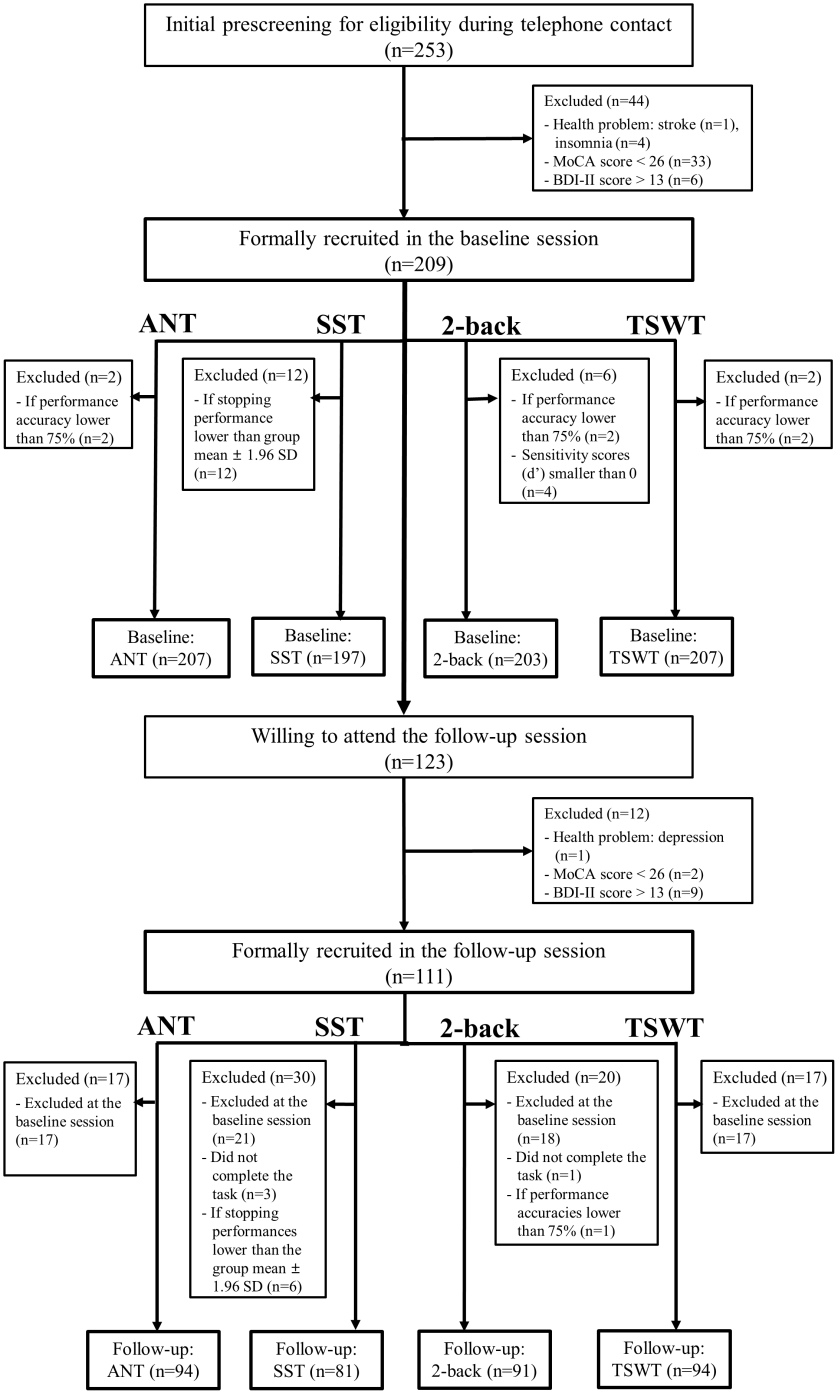
A flowchart indicates the screening criteria and numbers of participants for each of the four cognitive tasks (ANT, Attentional Network Test; SST, Stop-signal task; 2-back task; TSWT, Task-switching task) in the baseline and follow-up sessions. “n” denotes the number of participants either included or excluded. MoCA, Montreal Cognitive Assessment; BDI-II, Beck Depression Inventory–II; SD, standard deviation.

Participants’ demographic information and statistical results are shown in Table 1. The mean MoCA score was 27.78 ± 1.90 for all participants (*n* = 253) and 27.69 ± 1.85 for the subsample of 123 participants in the baseline session, and 28.93 ± 1.26 for the subsample of 123 participants in the follow-up session (*t* = −7.44, *p* < 0.00). The mean BDI-II score was 5.64 ± 4.72 for all participants (*n* = 253) and 4.92 ± 4.12 for the subsample of 123 participants in the baseline session, and 5.36 ± 5.91 for the subsample of 123 participants in the follow-up session (*t* = −0.87, *p* = 0.39; Table 1). Their manual motor function measured by GPT was 78.68 ± 20.71 (the average time spent by both hands) for all participants (*n* = 253) and 76.81 ± 14.90 for the subsample of 123 participants in the baseline session, and 77.81 ± 18.62 for the subsample of 123 participants in the follow-up session (*t* = −1.10, *p* = 0.27). Participants’ annual incomes and occupational classes between the baseline and follow-up sessions were not significantly different in the distribution (income: *p* = 0.89; occupational class: *p* = 0.12; Table 1), suggesting the homogeneity in the socioeconomic status between the two sessions. As mentioned, if we discovered that participants reported additional health problems (based on the Demographic and Health Status Questionnaire) that they did not self-disclose during the initial phone contact, if their MoCA or BDI-II scores did not pass the screening criteria, or if their performance on a computerized cognitive task did not pass the performance criteria of accuracy rate < 75% (including the incompletion of the task), these participants were further excluded. Therefore, the remaining number of participants for each cognitive task differed (described below) due to these screening criteria. Please see Figure 1 for the number and flow of participants from screening criteria for each of the four cognitive tasks. The remaining numbers of participants in the baseline session are *n* = 207 for ANT, *n* = 197 for SST, *n* = 203 for the 2-back task, and *n* = 207 for TSWT, and in the follow-up session are *n* = 94 for ANT, *n* = 81 for SST, *n* = 91 for the 2-back task, and *n* = 94 for TSWT (see also Table 2).

**Table 2:** Summary of the results for cognitive tasks in the baseline and follow-up test sessions.

The linear effect of age on attention and executive function	Baseline	Follow-up	Baseline-Follow-up (modified Brinley plot; based on the slope coefficient)
ANT-alerting	Decrease (*n* = 207)	Decrease (*n* = 94)	Decrease (*n* = 94)
ANT-orienting	No change (*n* = 207)	No change (*n* = 94)	Decrease (*n* = 94)
ANT-conflict	Increase (*n* = 207)	Increase (*n* = 94)	Increase (*n* = 94)
Stop-signal	Decrease (*n* = 197)	Decrease (*n* = 81)	Increase (*n* = 81)
2-back	Decrease (*n* = 203)	Decrease (*n* = 91)	Decrease (*n* = 91)
Task-switch	Increase (*n* = 207)	Increase (*n* = 94)	Increase (*n* = 94)

“Decrease” or “Increase” denoted in each cell indicates performance efficiency.

## Cognitive task performance: Cross-sectional data and longitudinal data for each of the cognitive tasks

### Attention network test

#### Cross-sectional data

##### Baseline session (*n* = 207)

###### Data trial exclusion and analysis

Before analysis, we discarded trials with timeouts (0.266%), incorrect responses (1.196%), and extremely fast responses (i.e., RT < 100 ms; 0.002%). Subsequent analyses were conducted on the log-transformed RTs.

###### Linear effects of age on each attentional network

**Figure 2:**
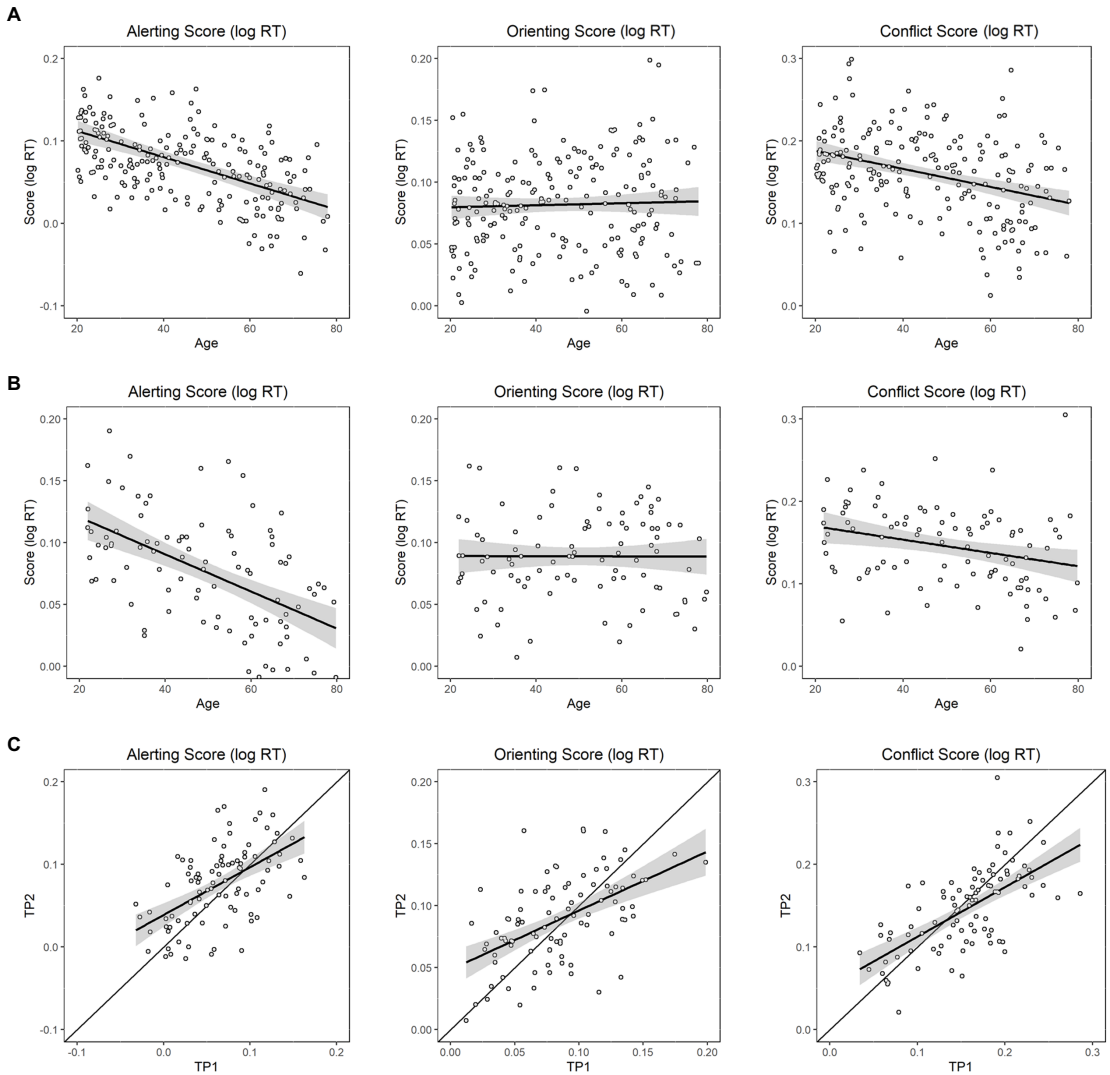
Linear effects of age on the efficiencies of the three attention networks. **(A)** In the baseline session, left panel: Effect on the efficiency of the alerting network; middle panel: Effect on the efficiency of the orienting network; right panel: Effect on the efficiency of the executive (conflict) network; **(B)** In the follow-up session, left panel: Effect on the efficiency of the alerting network; middle panel: Effect on the efficiency of the orienting network; right panel: Effect on the efficiency of the executive (conflict) network; **(C)** Modified Brinley plots for each attentional network (left panel: alerting; middle panel: orienting; right panel: conflict network). The X-axis denotes baseline, and Y-axis denotes follow-up. Scatterplots denote each data point for each individual. The best-fitting regression lines with confidence intervals are shown against the diagonal line.

Linear mixed-effects modeling showed that age had a significant main effect on the alerting network (i.e., subtracting the mean RT of the double-cue condition from the mean RT of the no-cue condition); older participants exhibited less of a benefit from alerting cues than younger participants (*p* < 0.001), suggesting a decreasing efficiency of the alerting network (Figure 2A, left panel). Second, age did not have a significant main effect on the orienting network (i.e., subtracting the mean RT of the spatial-cue condition from the mean RT of the center-cue condition; *p* = 0.52); participants with different ages exhibited similar benefits from spatial cues (Figure 2A, middle panel). Third, age had a significant main effect on conflict score (i.e., by subtracting the mean RT of all congruent conditions across all cue types from the mean RT of the incongruent conditions; p < 0.001), suggesting a paradoxically increasing efficiency of the conflict network with age (Figure 2A, right panel).

###### Nonlinear effects of age on each attentional network

Nonlinear mixed-effects modeling showed that age did not have significant main effects on any of the three ANT networks in the baseline session (all ps >0.05).

##### Follow-up session (*n* = 94)

###### Data trial exclusion and analysis

Before analysis, we discarded trials with timeouts (0.199%), incorrect responses (1.136%), and extremely fast responses (i.e., RT < 100 ms; 0.004%). Subsequent analyses were conducted on the log-transformed RTs.

###### Linear effects of age on each attentional network

Linear mixed-effects modeling showed that age likewise had a significant main effect on the alerting network in the follow-up session (*p* < 0.001), suggesting a decreasing efficiency of the alerting network (Figure 2B, left panel). Second, age did not have a significant main effect on the orienting network (*p* = 0.90), suggesting no change in the efficiency of the orienting network with age (Figure 2B, middle panel). Third, age had a significant main effect on conflict score (*p* = 0.01), showing a paradoxically increasing efficiency of the executive function reflected in conflict interference with age (Figure 2B, right panel).

###### Nonlinear effects of age on each attentional network

Nonlinear mixed-effects modeling in the follow-up session showed no age effect on any of the three ANT networks (all ps >0.05).

##### Longitudinal data

###### Modified Brinley plots for each attentional network

As mentioned, we applied a modified Brinley plot to interpret longitudinal changes. As shown in Figure 2C, many points are distributed above and below the diagonal line, indicating that the follow-up performance varied across participants. If we pooled all participants and modeled a best-fitting line across all points using linear regression models, we could observe a general trend for the follow-up pattern across participants. A fitting slope exceeding 1 suggests that the trend is larger for the follow-up measure relative to baseline, whereas a value smaller than 1 suggests that the overall trend is smaller for the follow-up measure relative to baseline. Based on this rationale, trends for a decreasing efficiency on alerting (Follow-up = 0.58*Baseline +0.04; slope ratio against 1, *p* < 0.00) and orienting (Follow-up = 0.48*Baseline +0.05; slope ratio against 1, p < 0.00) networks were observed, but paradoxically, increasing efficiency was observed for conflict (Follow-up = 0.6* Baseline +0.05; slope ratio against 1, p < 0.00) networks over a 1 ~ 2 year follow-up interval (Figure 2C).

### Stop-signal task

#### Cross-sectional data

##### Baseline session (*n* = 197)

###### Linear effects of age on inhibition

**Figure 3:**
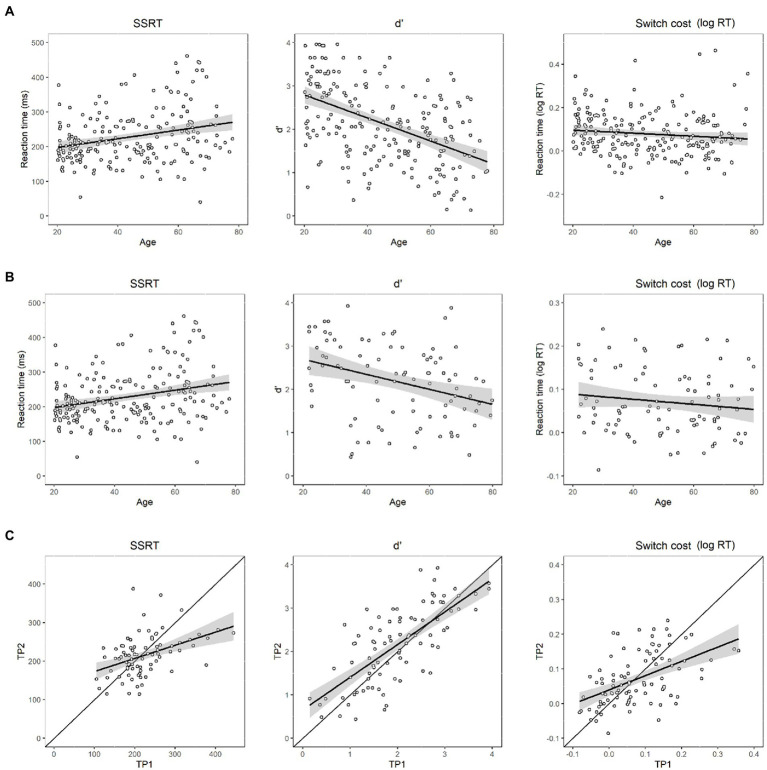
Linear effects of age on the efficiencies of the three tasks’ performance. **(A)** In the baseline session: left panel: Linear effects of age on stopping efficiency (SSRT: stop-signal reaction time); middle panel: Linear effects of age on 2-back sensitivity (d’); right panel: linear effects of age on switch cost (log-transformed RT); **(B)** In the follow-up session: left panel: Linear effects of age on stopping efficiency; middle panel: Linear effects of age on 2-back sensitivity (d’); right panel: linear effects of age on switch cost; **(C)** Modified Brinley plots for SSRT (left panel), d’ (middle panel), and switch cost (right panel). The *X*-axis denotes baseline, and *Y*-axis denotes follow-up. Scatterplots denote each data point for each individual. The best-fitting regression lines with standard deviations are shown against the diagonal line.

Linear regression models showed that age had a significant main effect on SSRT; older participants exhibited longer SSRTs than younger participants (*p* < 0.001), suggesting a decreased efficiency of inhibitory function (Figure 3A, left panel).

###### Nonlinear effects of age on inhibition

A nonlinear effect of age on SSRT was not observed in the baseline session (*p* > 0.05).

##### Follow-up session (*n* = 81)

###### Linear effects of age on inhibition

In the follow-up session, the results for SSRT likewise showed a significantly decreased efficiency of inhibitory function (*p* < 0.02) for older adults (Figure 3B, left panel).

###### Nonlinear effects of age on inhibition

No nonlinear effect of age on SSRT was observed in the follow-up session (*p* > 0.05).

##### Longitudinal data

##### Modified Brinley plots for inhibition

The modified Brinley plot for baseline follow-up on SSRT is shown in the left panel of Figure 3C. Efficiency in SSRT increased from baseline to follow-up (Follow-up = 0.34*Baseline +139.00; slope ratio against 1, *p* < 0.00).

### 2-back task

#### Cross-sectional data

##### Baseline session (*n* = 203)

###### Data trial exclusion and analysis

Before analysis, we discarded trials with no response (2.28%) and extremely fast responses (i.e., RT < 100 ms; 0.07%).

###### Linear effects of age on working memory updating

Linear regression models showed that age had a significant main effect on 2-back d’ in the baseline session; older participants exhibited a smaller d’ than younger participants (*p* < 0.001), suggesting a decreasing efficiency of updating function (Figure 3A, middle panel).

###### Nonlinear effect of age on working memory updating

No nonlinear effect of age was observed on working memory updating in the baseline session (*p* > 0.05).

##### Follow-up session (*n* = 91)

###### Data trial exclusion and analysis

Before analysis, we discarded trials with no response (1.31%) and extremely fast responses (i.e., RT < 100 ms; 0.124%).

###### Linear effects of age on working memory updating

As in the baseline session, linear regression models also showed that age had a significant main effect on the 2-back d’ in the follow-up session; older participants exhibited smaller d’ than younger participants (*p* < 0.01), suggesting a decreasing efficiency of updating function (Figure 3B, middle panel).

###### Nonlinear effect of age on working memory updating

No nonlinear effect of age on working memory updating was observed in the follow-up session (*p* > 0.05).

##### Longitudinal data

###### Modified Brinley plots for working memory updating

As shown in the middle panel of Figure 3C, many points are distributed both above and below the diagonal line, indicating that the follow-up performance varied across participants. If we pooled all participants and modeled a best-fitting line across all points, we could observe a general trend for the follow-up pattern across participants. The result shows that the ratio of the best fitting regression line was significantly smaller than 1 (Follow-up = 0.76*Baseline +0.65; slope ratio against 1, *p* < 0.00), suggesting a decrease in memory updating ability from baseline to follow-up.

### Task-switching task

#### Cross-sectional data

##### Baseline session (*n* = 207)

###### Data trial exclusion and analysis

Before analysis, we discarded trials with timeouts (0.200%), incorrect responses (3.738%), and extremely fast responses (i.e., RT < 100 ms; 0.162%). Subsequent analyses were conducted on the log-transformed RTs.

###### Linear effects of age on switching ability

The linear mixed-effect model showed a significant age effect on switch cost, yet with a paradoxical direction: the switch cost decreased significantly with age (*p* < 0.001), suggesting increased efficiency in shifting ability with age (Figure 3A, right panel).

###### Nonlinear effects of age on switching ability

No significant nonlinear effect of age on switch cost was observed in the baseline session (*p* > 0.05).

##### Follow-up (*n* = 94)

###### Data trial exclusion and analysis

Before analysis, we discarded trials with timeouts (0.0973%), incorrect responses (3.1854%), and extremely fast responses (i.e., RT < 100 ms; 0.6161%). Subsequent analyses were conducted on the log-transformed RTs.

###### Linear effects of age on switching ability

The linear mixed-effect model showed a significant age effect on switch cost (*p* = 0.02), yet with a paradoxical direction: the switch cost decreased significantly with age, suggesting increased efficiency in shifting ability with age (Figure 3B, right panel).

###### Nonlinear effects of age on switching ability

No significant nonlinear effect of age on switching costs was observed in the follow-up session (*p* > 0.05).

##### Longitudinal data

###### Modified Brinley plots for switching ability

The modified Brinley plot for local switch cost is shown in the right panel of Figure 3C. The linear regression model showed significantly increasing efficiency from baseline to follow-up (Follow-up = 0.41*Baseline +0.04; slope ratio against 1, p < 0.00).

## Discussion

This study aimed to investigate age-related effects on attention and EF across the adult lifespan. We employed cross-sectional and longitudinal approaches to minimize cohort effects and tap into individual differences in their cognitive aging trajectories. We employed various attention and EF tasks, including ANT, SST, 2-back task, and TSWT, in the study. The current analysis of cross-sectional data showed that older adults exhibited decreased efficiency in alerting, stopping ability, and working memory updating in both the baseline and follow-up sessions (see Table 2 for a summary of results). Conversely, older adults exhibited paradoxically increased efficiency in conflict control and switching abilities in both baseline and follow-up sessions and showed no change in orienting efficiency with age.

The current cross-sectional results of the ANT accorded with those reported by Veríssimo et al. (2022), except for the orienting efficiency, for which they observed decreased efficiency in the alerting network with age (see also Festa-Martino et al., 2004) but increased efficiency in both the orienting and conflict control networks with age. Two major differences exist between the two studies: (1) Veríssimo et al.’s (2022) participants were aged between 58 and 98 years, whereas the current study covered a wider age range, i.e., 20–78 yrs. Furthermore, (2) Veríssimo et al.’s (2022) sample size was 702, whereas the current study’s sample sizes were 207 for the baseline session and 94 for the follow-up session. Nevertheless, despite these major differences, the current findings on the ANT performance for both the baseline and follow-up sessions generally replicated the findings reported by Veríssimo et al. (2022), and the statistical power of the current study exceeded 0.90 (G*Power ver. 3.1.9.7). In addition, the lack of change in the orienting network observed in this study was consistent with some previous studies that suggest that older adults could benefit as much as younger adults from physical or symbolic cues that direct attention to the likely spatial location (e.g., Greenwood and Parasuraman, 1994; Kramer and Strayer, 2001; Fernandez-Duque and Black, 2006; Jennings et al., 2007; Zhou et al., 2011). Nevertheless, the lack of change (the current study) or increase (Veríssimo et al., 2022) in orienting efficiency in the orienting network was based on cross-sectional data.

Despite being consistent with the findings of Veríssimo et al. (2022), the increased efficiency for the conflict network reported herein may appear to be inconsistent with the findings of some previous studies (e.g., Mahoney et al., 2010; Zhou et al., 2011; Lu et al., 2016; Dash et al., 2019). This finding also contradicts long and widely believed cognitive control deficits in aging (Hasher and Zacks, 1988; Craik et al., 1990; Salthouse, 1990; Moscovitch and Winocur, 1992; West, 1996). We suspect that the paradoxical phenomenon of increased efficiency on conflict scores for older adults observed herein might be partly related to the increased perceptual load (Lavie and Tsal, 1994; Lavie, 1995, 2005, 2010; Murphy et al., 2016) for older adults that are often seen in a flanker task (Moss et al., 2020). Alternatively, this result might be related to the strategy used by older adults, as reported by a series of studies by Hsieh and colleagues (Hsieh et al., 2012; Hsieh and Fang, 2012; Hsieh and Lin, 2014). Specifically, their event-related studies have shown that older adults could be just as capable as younger adults in resolving flanker interference by adopting different strategies (e.g., increased attention to the target) to compensate for their deficiencies. Third, the paradoxically increased efficiency in age-related conflict control ability may be explained by the lifelong experience account suggested by Veríssimo et al. (2022). As mentioned, Veríssimo et al. (2022) observed that older adults paradoxically benefited more from spatial cues (i.e., orienting network) and were less interfered with by flanker distractions (i.e., conflict network). To account for the paradoxical phenomena, Veríssimo et al. (2022) proposed a neurocognitive account of age effects on the three attentional networks, suggesting that age-related efficiency changes are explained by a combination of various neurobiological-based mechanisms, including declines, maintenance, compensation, and reserve, in which neural declines can be counteracted by the latter three mechanisms, particularly those gained from lifelong experience. According to this account, Veríssimo et al. (2022) suggested that the orienting and executive networks involved some processing strategies that could benefit from practice and training, whereas alerting involved more basic processes of vigilance and preparedness, which were less sensitive to practice and training (Posner and Petersen, 1990; Posner and Rothbart, 2007; Petersen and Posner, 2012).

However, as mentioned in the Introduction, EF consists of various components, such as inhibition, updating, and switching (e.g., Miyake et al., 2000); furthermore, differences even exist within a single component: the inhibition component consists of different forms, such as inhibition of prepotent responses, inhibition of ongoing responses, and the ability to resist distractor interference (e.g., Barkley, 1997; Rey-Mermet and Gade, 2018). Therefore, the conflict control measured by the ANT is only one type of inhibition component. To provide a more comprehensive pattern of executive functions in aging, this study also incorporated different EF tasks, such as SST, 2-back, and TSWT. The cross-sectional data obtained in this study indeed showed age-related deficits in other types of executive functions, such as stopping ability (measured by a stop-signal task) and memory updating sensitivity (d’ measured by a 2-back task). However, no age-related deficit was identified in the switching ability. Instead, a paradoxically increased efficiency was identified in this dimension. Therefore, the results on these various types of tasks obtained in this study accord with our prediction that only some attention dimensions and EF declined with age, such as alerting, stopping ability, and memory updating, which showed a significant decline. Furthermore, the current findings, along with other previous data, contradict theories that postulated general age-related declines in attention and EF and general age-related inhibition deficits, as proposed by Hasher and Zacks (1988) and Hasher et al. (1991, 2007). The current cross-sectional results also agreed with those reported by review articles, such as Verhaeghen (2011, 2014), Wasylyshyn et al. (2011), and Rey-Mermet and Gade (2018), suggesting specific but not generalized age-related deficits in executive control functions. Furthermore, these results could be linked to the cognitive reserve theory proposed by Stern (2002, 2009) and Steffener et al., 2014: they suggested that some individuals may maintain longer and better functioning than others due to differences in life experiences. Thus, observing an individual longitudinally may provide a more informed picture of aging trajectories.

A novelty of this study was to also examine the longitudinal follow-up data by means of the modified Brinley plot, and the results showed that not all findings on the cross-sectional data were replicated in the longitudinal data: only alerting and memory updating persistently showed decreased efficiency with aging, and conflict control and switching persistently showed increased efficiency with aging. Conversely, the orienting network and stopping no longer showed results similar to those in the cross-sectional data. That is, the discrepancies between the results analyzed from the cross-sectional and longitudinal approaches regarding the age effect were in the orienting network, and stopping efficiencies. Among these discrepancies, the most surprising finding is that stopping showed a deficit based on the cross-sectional data but a paradoxically increased efficiency in the follow-up data. Most previous research has reported an age-related decline in these two abilities. The decreased stopping ability for older adults observed in the cross-sectional data in this study is in line with several prior studies showing age-related deficit stop-signal inhibition deficits (e.g., Kramer et al., 1994; Bedard et al., 2002; van de Laar et al., 2011; Kleerekooper et al., 2016). However, other previous studies showed no age-related decline (e.g., Kray et al., 2009) or only a specific deficit (e.g., Anguera and Gazzaley, 2012; Hsieh and Lin, 2017). Furthermore, the longitudinal results paradoxically obtained herein showed an opposite direction, i.e., an increased efficiency from baseline to follow-up.

One possibility for the discrepancies in stopping age-related effects between cross-sectional and longitudinal results in the current study might be that the cohort effect exaggerates age deficits in the cross-sectional data, as often reported in the literature. Alternatively, the discrepancy may be due to the practice effect on the follow-up data because the follow-up interval in the current study was only 1 ~ 2 years, which is considered short. As mentioned above, Veríssimo et al. (2022) suggested that orienting and executive improvements can be largely explained by lifelong experience with these networks. Therefore, we might extend Veríssimo et al.’s (2022) hypothesis to the stopping function. Accordingly, the lack of decline in stopping efficiency in the longitudinal data observed herein might also be due to the fact that this ability could potentially benefit from practice and training (i.e., 1 ~ 2 years of follow-up retest), like the orienting and conflict networks. Based on this speculation, we further suggested that if the practice could successfully overcome the possible aging deficit over a period of 1 ~ 2 years, the abilities of older adults can still be comparable to those of young adults after practicing. This speculation may offer implications for developing clinical intervention programs to delay the trajectories of cognitive aging. Nevertheless, individual differences may exist in practice and experience. The current longitudinal data show that the scatterplot points for the SSRT in the modified Brinley plots were distributed in a wide range in the x-y plane. However, most of these data points might be within the reliable change interval. Likewise, the discrepant findings regarding the orienting network showed no change with age cross-sectionally but a significant decrease in efficiency with aging longitudinally. Thus, individual differences might also affect this dimension since the scatterplot points showed a similarly wide distribution in the modified Brinley plot. As such, individual longitudinal differences remain an important issue for future aging research. Furthermore, whether a longer follow-up period (i.e., longer than 2 years) will result in similar findings regarding stopping function as currently observed also warrants future research.

Regarding the task-switching performance for older adults observed in this study, the literature has similarly found little concrete evidence for age-related differences in local switching costs. For example, the literature has shown unequivocal results on local switching costs, including either an age-related increase (yet mostly observed with pathological aging participants, such as mild cognitive impairment or Alzheimer’s disease; e.g., Belleville et al., 2008), a decrease (with normal aging participants; e.g., Whitson et al., 2014), and the results from the present study), or no difference across different age groups (with normal aging participants; e.g., Kray et al., 2005; Reimers and Maylor, 2005). Therefore, the current cross-sectional results align with previous results reported by normal aging participants showing no age-related decline in task switching among various executive functions (Verhaeghen, 2011, 2014). Furthermore, the current results also showed increased switching efficiency cross-sectionally and longitudinally, suggesting that an individual is still capable of shifting different tasks during aging, perhaps due to lifelong experience and practice.

Finally, as mentioned, only alerting and memory updating showed consistently decreased efficiency with aging in this study. The literature has commonly shown an age-related decline in the ability to use alerting cues (e.g., Festa-Martino et al., 2004; Jennings et al., 2007; Gamboz et al., 2010; Zhou et al., 2011) and attributed such a deficit to changes in noradrenergic signaling (e.g., Lohr and Jeste, 1988; Ferrari and Magri, 2008; Robertson, 2013). Furthermore, Thiel et al. (2004) observed that the alerting effect was associated with increased extrastriate activations (Thiel et al., 2004). Both Veríssimo et al.’s (2022) study and the current results align with the literature showing that alerting deficits are most vulnerable to age differences/changes. Alerting ability is a crucial survival skill for human beings. Therefore, strategies to prevent and improve older people’s alertness is an important practical issue in aging research. Some researchers have indicated that not all forms of alerting cues are vulnerable to age. For example, Erel et al. (2020) indicated that while older adults benefited less from visual alerting cues than younger adults, they nevertheless used auditory alerting cues equally well as younger adults. Furthermore, they found that if visual signals lasted longer, older adults could still gain benefits from visual alerting cues (Erel et al., 2020). Therefore, the implications from the current study and prior research for daily life practice are to add more auditory cues or longer visual cues to the environment (e.g., adding auditory cues or more prominent visual cues in traffic designs; see also Erel et al., 2020) since an aging society is currently inevitable worldwide. These implications require future research to confirm. Likewise, working memory is also a critical component of complex cognition (e.g., it facilitates planning, comprehension, reasoning, and problem-solving, etc.), and has long been known to be sensitive to age (Schmiedek et al., 2009; Verhaeghen and Zhang, 2013; Loosli et al., 2014; Bopp and Verhaeghen, 2020). Therefore, strategies to prevent and improve people’s working memory, especially the memory updating ability, is also an important practical issue in aging research (for a review, see Pappa et al., 2020).

## Limitations of the study

Some issues warrant discussion before closing. First, although this study recruited more than 200 participants (i.e., *n* = 253 for the baseline session) for the baseline session, the number of participants (*n* = 123 for the follow-up session) was largely reduced due to the dropout rate and screening criteria. Nevertheless, the reduced number for the follow-up session (*n* = 81 ~ 94) retained sufficient statistical power (ranging between 0.87–1.0 for cognitive tasks calculated by G*power ver. 3.1.9.7). Second, the short follow-up interval (i.e., 1 ~ 2 years) is likely to alleviate the aging effect due to the practice effect. Performance on cognitive tests improves following the repeated administration of tests, which has long been known in longitudinal studies (Wilson et al., 2006). We acknowledged this inevitable practice effect (Goldberg et al., 2015) and directly tested this possible practice effect by adding the variable of interval length (1 ~ 2 years.) into linear and nonlinear mixed effect models. However, the results of these models showed neither a main effect of interval length nor its interactions with other variables (such as age and condition). Hence, although the practice effect for the retest is inevitable, the current null result of interval length suggested that the changes in performance from baseline to follow-up cannot be attributed to the practice effect. Nevertheless, methods to minimize the practice (retest) effect for follow-up studies require further endeavors, despite the lack of consensus on the best method after over a century of research. Third, this study only followed participants once. Informed longitudinal follow-up studies should acquire additional follow-up sessions (frequency) (Pullenayegum et al., 2021). Future studies are encouraged to acquire more follow-up sessions.

## Conclusion

To conclude, converging the current cross-sectional and longitudinal findings showed that among attention and EF, the alerting and memory updating function showed the most robust deficit with age (cross-sectional) and aging (longitudinal). Although stopping efficiency was also vulnerable to age differences (i.e., cross-sectional data), these abilities did not intraindividually decline significantly over 2 years. However, individual differences are not negligible and warrant future investigation. The contribution of the current study is to provide practical implications for future developing strategies to prevent age-related declines for the most sensitive attention and EF functions.

## Data availability statement

The data analyzed in this study are available upon request. Requests to access these datasets should be directed to SH, psyhsl@ncku.edu.tw.

## Ethics statement

The studies involving human participants were reviewed and approved by the study protocol was approved by the Human Research Ethics Committee (No. 104–004) at National Cheng Kung University, Tainan, Taiwan. The patients/participants provided their written informed consent to participate in this study.

## Author contributions

SH designed the analysis protocol, applied for the research funding, designed the research protocol, and supervised data analyses. E-HC analyzed the data, drafted the results sections, and generated figures. All authors contributed to the article and approved the submitted version.

## Funding

The authors thank the National Science and Technology Council (NSTC), originally known as the Ministry of Science and Technology (MOST), Taiwan (ROC) of the Republic of China, for financially supporting this research (grant nos. 104-2410-H-006-021-MY2, 106-2410-H-006-031-MY2, 108-2321-B-006-022-MY2, 108-2410-H-006-038-MY3, 109-2923-H-006-002-MY3, 110-2321-B-006-004, 111-2321-B-006-008, and 111-2410-H-006-114). In addition, this research was supported in part by Higher Education Sprout Project, Ministry of Education to the Headquarters of University Advancement at National Cheng Kung University (NCKU) (grant numbers: D111-F2903; D111-F2909; R111-B013). The authors also thank Mind Research and Imaging Center (MRIC) at National Cheng Kung University for consultation and instrument availability.

## Conflict of interest

The authors declare that the research was conducted in the absence of any commercial or financial relationships that could be construed as a potential conflict of interest.

## Publisher’s note

All claims expressed in this article are solely those of the authors and do not necessarily represent those of their affiliated organizations, or those of the publisher, the editors and the reviewers. Any product that may be evaluated in this article, or claim that may be made by its manufacturer, is not guaranteed or endorsed by the publisher.

## Footnotes

^1^Note: Physical activity was evaluated from Level 1 (rarely engage in physical activity) to Level 5 (participate in aerobic exercise, more than 3 h per week).

^2^In this test, participants were asked to place as many cylindrical metal pegs into one of 25 holes in a pegboard as quickly as possible, by using once with the left hand and once with the right hand. The total time to complete the task is used as an index of GPT performance for each hand. Shorter total completion time (in seconds) indicates better performance. Here, we averaged the performance of the two hands.

^3^We classified the annual income into four classes: Low income: under 120,000 NTD per year; Middle income: 120,000 ~ 480,000 NTD per year; High income: above 480,000 NTD per year.

^4^We classified the occupational class into four types: No job: student, retired, housekeeping, etc.; Light workload: Office worker such as manager, designer, computer engineer, clerical work; Moderate workload: Sales staff, long-term standing, and walking needs such as business, teachers, doctors, nurses; Heavy workload: Manual labor work such as laborer.

^5^Note, we only tested on those participants (n = 123) who attended both the baseline and follow-up sessions.
